# The histone chaperone protein Nucleosome Assembly Protein-1 (hNAP-1) binds HIV-1 Tat and promotes viral transcription

**DOI:** 10.1186/1742-4690-5-8

**Published:** 2008-01-28

**Authors:** Chiara Vardabasso, Lara Manganaro, Marina Lusic, Alessandro Marcello, Mauro Giacca

**Affiliations:** 1 Molecular Medicine Laboratory, International Centre for Genetic Engineering and Biotechnology (ICGEB), Padriciano 99, 34012 Trieste, Italy; 2 Molecular Virology Laboratory, International Centre for Genetic Engineering and Biotechnology (ICGEB), Padriciano 99, 34012 Trieste, Italy

## Abstract

**Background:**

Despite the large amount of data available on the molecular mechanisms that regulate HIV-1 transcription, crucial information is still lacking about the interplay between chromatin conformation and the events that regulate initiation and elongation of viral transcription. During transcriptional activation, histone acetyltransferases and ATP-dependent chromatin remodeling complexes cooperate with histone chaperones in altering chromatin structure. In particular, human Nucleosome Assembly Protein-1 (hNAP-1) is known to act as a histone chaperone that shuttles histones H2A/H2B into the nucleus, assembles nucleosomes and promotes chromatin fluidity, thereby affecting transcription of several cellular genes.

**Results:**

Using a proteomic screening, we identified hNAP-1 as a novel cellular protein interacting with HIV-1 Tat. We observed that Tat specifically binds hNAP1, but not other members of the same family of factors. Binding between the two proteins required the integrity of the basic domain of Tat and of two separable domains of hNAP-1 (aa 162–290 and 290–391). Overexpression of hNAP-1 significantly enhanced Tat-mediated activation of the LTR. Conversely, silencing of the protein decreased viral promoter activity. To explore the effects of hNAP-1 on viral infection, a reporter HIV-1 virus was used to infect cells in which hNAP-1 had been either overexpressed or knocked-down. Consistent with the gene expression results, these two treatments were found to increase and inhibit viral infection, respectively. Finally, we also observed that the overexpression of p300, a known co-activator of both Tat and hNAP-1, enhanced hNAP-1-mediated transcriptional activation as well as its interaction with Tat.

**Conclusion:**

Our study reveals that HIV-1 Tat binds the histone chaperone hNAP-1 both in vitro and in vivo and shows that this interaction participates in the regulation of Tat-mediated activation of viral gene expression.

## Background

Efficient packaging of DNA in a highly organized chromatin structure inside the cell is one of the most remarkable characteristics of all eukaryotic organisms. Chromatin assembly and disassembly are dynamic biological processes that increase chromatin fluidity and regulate the accessibility of the genome to all DNA transactions, including transcription, DNA replication and DNA repair. The basic structural unit of eukaryotic chromatin is the nucleosome, formed by the wrapping of DNA around an octamer of core histone proteins. By restricting the access to DNA-binding factors and impeding elongation by RNA polymerase II (RNAPII), the nucleosome is not only a structural unit of the chromosome, but perhaps the most important regulator of gene expression (for recent reviews, see refs. [1,2]). Chromatin structure is modulated by the covalent modifications of the N-termini of the core histones in nucleosomes and by the action of ATP-dependent chromatin remodeling complexes. In particular, histone acetylation at the promoter of genes, mediated by histone acetyltransferases (HATs), has been shown to be necessary, albeit not sufficient, for transcriptional activation [2,3].

Chromatin assembly is a stepwise process which requires histone chaperones to deposit histones on forming nucleosomes (reviewed in refs. [4-7]). The Nucleosome Assembly Protein-1 (NAP-1) is one of the major histone chaperones involved in this process. This factor belongs to the NAP family of proteins, which is characterized by the presence of a NAP domain [8]. NAP-1 is conserved in all eukaryotes from yeast to humans [9-12], and is responsible for the incorporation of two histone H2A-H2B dimers to complete the nucleosome (reviewed in ref. [7]). The protein acts as a nucleo-cytoplasmic shuttling factor that delivers H2A-H2B dimers from cytoplasm to the chromatin assembly machinery in the nucleus [13]. In addition, NAP-1 has been involved in the regulation of cell-cycle progression [14-16], incorporation and exchange of histone variants [17-19], and promotion of nucleosome sliding [20].

Most relevant to the regulation of gene expression, the chromatin-modifying activity of histone chaperones also facilitates transcription. In particular, recent information suggests that HAT complexes as well as ATP-dependent chromatin remodeling complexes cooperate with histone chaperones in altering chromatin structure during transcriptional activation [21-24]. In addition, NAP proteins have been reported to interact with the histone acetyltransferase (HAT) and transcriptional coactivator p300/CBP [25-27], suggesting that NAPs may augment activation by all the transcription factors that use p300/CBP as a co-activator. Accordingly, a yeast two-hybrid screen revealed that hNAP-1 forms a complex with the HPV E2 transcription factor, and a complex formed by hNAP-1, E2 and p300 proved able to activate transcription in vitro [28].

One of the promoters that show exquisite sensitivity to regulation by chromatin structure and its modifications is the long terminal repeat (LTR) of the Human Immunodeficiency Virus type 1 (HIV-1) (reviewed in ref. [29]). Following infection of susceptible cells, the HIV-1 provirus becomes integrated into the host genome and, for still poorly understood reasons, the LTR promoter enters a latent state and becomes silenced by chromatin conformation [29,30]. Independent of the site of integration, two distinct nucleosomes are precisely positioned in the 5' LTR, separated by a nuclease-hypersensitivity region containing the enhancer and basal promoter elements [31-34]. Genomic footprinting experiments performed in either activated or latently infected cells have revealed that most of the critical protein-DNA interactions in the promoter region are preserved, independent from the LTR activation state [35,36]. This observation first indicated that the transcriptional activation of the integrated LTR is not primarily restricted by DNA target site accessibility, but occurs through the modulation of chromatin conformation. Indeed, Nuc-1, which is positioned near the viral mRNA start site, appears to exert a repressive role on transcription; this nucleosome becomes remodelled when HIV-1 transcription is activated [37,38]. Which are the factors involved in chromatin remodelling during transcriptional activation, besides the recruitment of several HATs [39], is a still poorly addressed question.

One of the key factors involved in transcriptional activation of the provirus is the HIV-1 Tat protein, a highly unusual transactivator that binds an RNA element (TAR) positioned at the 5' end of the primary proviral transcript [40]. Tat activates HIV-1 transcription by promoting the assembly of transcriptionally active complexes at the LTR by multiple protein-protein interactions. Over the last few years, a number of cellular proteins have been reported to interact with Tat and to mediate or modulate its activity. Among these interacting partners, a major role can be ascribed to the P-TEFb complex [41-43] and to several cellular HATs, including p300/CBP, P/CAF and GCN5 [44-47]. P-TEFb promotes processive transcription by phosphorylating the RNAPII carboxy-terminal domain (CTD) [48,49], while HATs induce the activation of chromatinized HIV-1 LTR through the acetylation of histones [39]. Of interest, optimal Tat-mediated activation of viral gene expression also requires the function of ATP-dependent chromatin-remodelling complexes [50].

In this work we address the issue of identifying novel cellular interactors of Tat through a proteomic screening. We identify human NAP-1 as a major Tat partner and show that the interaction between the two proteins is important for Tat-mediated transcriptional activation and for efficient viral infection.

## Results

### Identification of cellular factors binding to HIV-1 Tat by proteomic analysis

**Figure 1:**
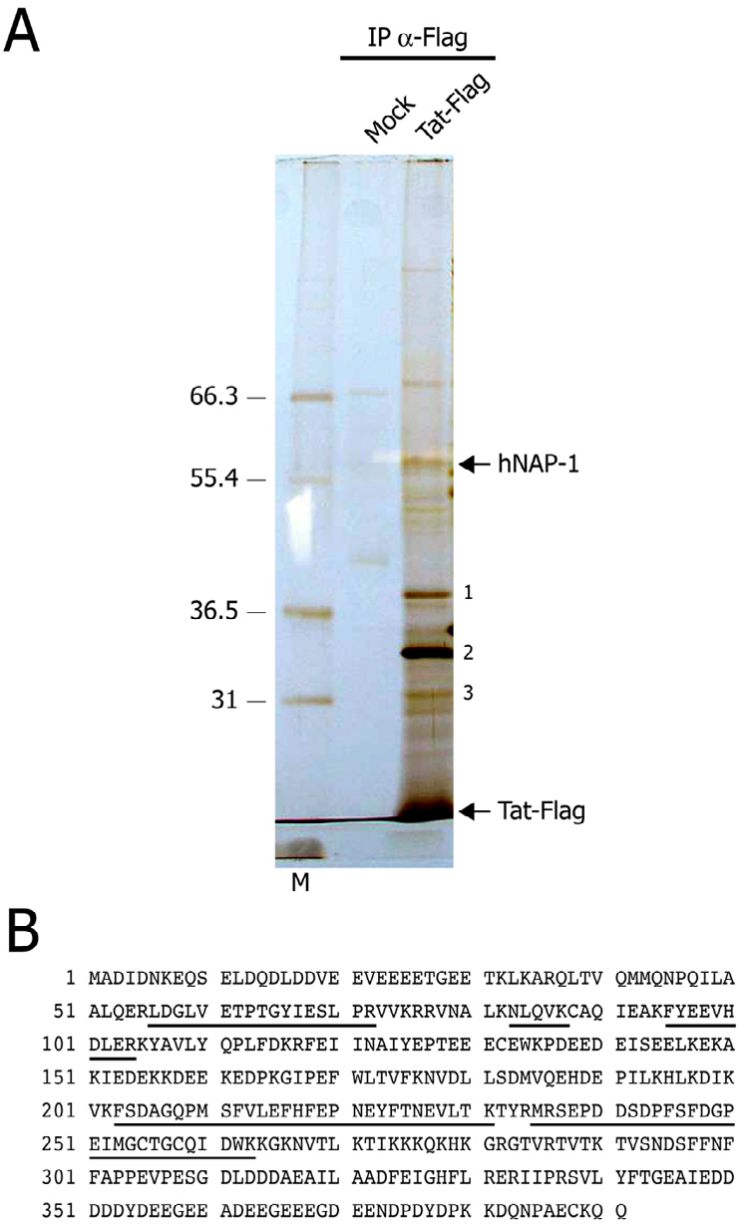
**Identification of Tat-interacting proteins by mass spectrometry. A**. Flag-immunoprecipitated material from Tat-Flag- and mock-transfected HEK 293T cells was resolved by 6–10% gradient SDS-PAGE gel, followed by silver staining. Protein bands present exclusively in the sample transfected with Tat-Flag were excised from the gel and their identification attempted by ESI-MS/MS. The identified proteins, in addition to hNAP-1 and Tat-Flag, are indicated (1: B23/nucleophosmin; 2: pre-mRNA splicing factor SF2p32 – Tat-associated protein TAP; 3: ribosomal protein S4). **B. **Amino acid sequence of the human NAP-1 protein (locus NP_631946) – 391 aa. The underlined amino acid sequences correspond to peptides obtained from MS/MS analysis of three independent preparations (P = 7.8 × 10^-19^).

With the aim of identifying cellular partners of HIV-1 Tat through a proteomic approach, we used an expression vector encoding the open reading frame of full length Tat (101 aa) fused with a C-terminal Flag tag. This epitope-tagged version of Tat was active in HIV-1 LTR transactivation similar to the wild type protein (data not shown). Extracts from HEK 293T cells transfected with Flag-Tat101, as well as from mock-transfected cells, were immunoprecipitated with M2 Flag antibody conjugated to agarose beads. Affinity purified Tat-Flag protein and co-purifying cellular factors were subsequently eluted with an excess of Flag peptide, run on a 6–15% gradient SDS-PAGE gel and stained with silver stain (Figure 1). Individual bands that were apparent only in the sample from Tat-Flag transfected cells were excised and their identification attempted by ESI-MS/MS (Electrospray tandem Mass Spectrometry) analysis of peptides obtained after trypsin digestion. Five bands were unequivocally identified, as shown in Figure 1. One corresponding to Tat-Flag itself; B23/nucleophosmin, a nucleolar protein possibly associated with ribosome assembly and/or transport [51]; the p32 protein, an inhibitor of the ASF/SF2 splicing regulator [52], also known as Tat-associated protein (TAP) [53,54]; ribosomal protein S4 and the histone chaperone NAP-1 (Nucleosome Assembly Protein-1). The proteomic analysis was repeated and the results were also confirmed by sequencing proteins directly from the Flag beads, rather than from gel-excised bands.

Since overexpressed Tat is known to accumulate in the nucleoli, probably due to its unspecific RNA binding capacity, and given the observation that the same proteomic assay resulted in the identification of a number of other ribosomal proteins when performed in the absence of RNase (data not shown), no further work was performed on the B23/nucleophosmin and ribosomal S4 proteins. In this respect, other investigators have already shown that Tat binds B23/nucleophosmin when both proteins are overexpressed [55] and that B23/nucleophosmin protein is required for Tat nucleolar localization but not for promoter transactivation [56]. The rest of our research was therefore focused on the characterization of the hNAP-1/Tat interaction.

### HIV-1 Tat interacts with hNAP-1 in vivo

**Figure 2:**
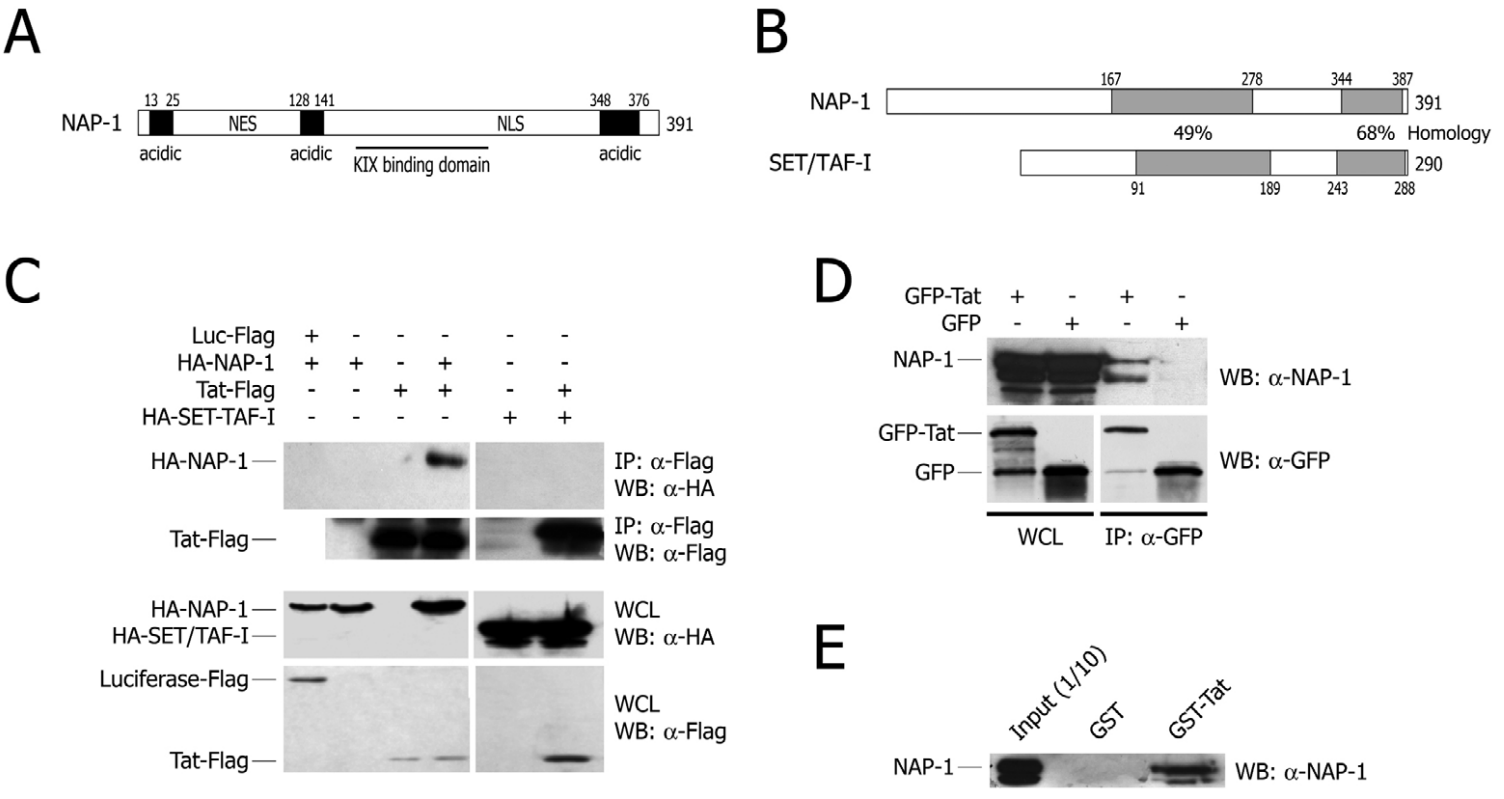
**Co-immunoprecipitation of Tat with transfected and endogenous hNAP-1. A**. Schematic representation of hNAP-1 structure. The acidic domains of the protein are shown by black boxes, with the indication of their boundary amino acids. The localization of nuclear export and nuclear localization signals (NES and NLS respectively) are indicated. **B. **Schematic representation of the regions of amino acid homology between hNAP-1 and hSET/TAF-I. **C. **Co-immunoprecipitation of transfected hNAP-1 with Tat. The plasmids indicated on top of the figure were transfected into HEK 293T cells. The upper two panels show western blots with the indicated antibodies after immunoprecipitation using an anti-Flag antibody; the lower two panels show western blotting controls from whole cell lysates (WCL) from transfected cells to show the levels of expression of the transfected proteins. **D. **Co-immunoprecipitation of endogenous hNAP-1 with Tat. The experiment was performed by transfecting HEK 293T cells with plasmids encoding GFP-Tat or GFP alone, followed by co-immunoprecipitation with anti-GFP antibody. GFP-Tat retains full transcriptional and trafficking capacities as wt Tat [69, 74, 75]. **E. **GST-pulldown experiment using GST-Tat and HEK 293T whole cell lysates. GST-Tat, but not control GST protein, pulled down endogenous hNAP-1.

A schematic representation of hNAP-1 is shown in Figure 2A. The protein has 391 amino acids, contains three acidic domains and has a long KIX-binding domain. This domain and the C-terminal acidic domain are very conserved in other members of the NAP family of histone chaperones, including SET-TAF-I (47% and 68% amino acid homology in the two regions respectively [57,58]; Figure 2B).

The interaction between HIV-1 Tat and hNAP-1 was confirmed by co-immunoprecipitation analysis. When expression vectors for Tat-Flag and for an N-terminal HA-tagged version of hNAP-1 (HA-NAP-1) were transfected into HEK 293T, HA-NAP-1 was co-immunoprecipitated with Tat using anti-Flag antibody (Figure 2C). The specificity of interaction of the two proteins is underlined by the observation that no co-immunoprecipitation was observed when Tat was co-expressed with HA-hSET/TAF-I, despite its sequence homology with hNAP-1 (Figure 2C).

Tat was also found to bind endogenous hNAP-1. As shown in Figure 2D, an anti-GFP antibody was able to precipitate endogenous hNAP-1, as detected with an anti-hNAP-1 antibody, from extracts of cells transfected with GFP-Tat but not from extracts of cells transfected with control GFP.

Finally, a bacterially expressed and purified GST-Tat recombinant protein was also able to pull-down endogenous hNAP-1 from a HEK 293T cell extract (Figure 2E).

### Binding domain analysis

**Figure 3:**
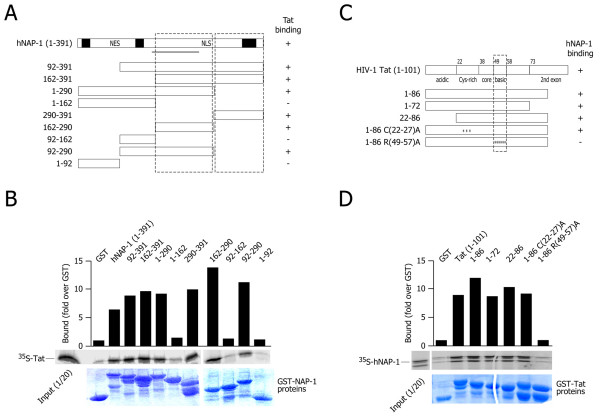
**Mapping of hNAP-1 and Tat interacting domains. A**. Schematic representation of hNAP-1 protein and of its deletion mutants obtained as GST fusion proteins. The capacity of binding to Tat – see experiment in panel B – is indicated on the right side of each mutant. The two dotted boxes indicate the hNAP-1 domains interacting with Tat. **B. **Representative GST pulldown experiment using the indicated hNAP-1 mutants and radiolabelled Tat101 protein. The autoradiography shows the amount of Tat binding to each mutant; the histogram on top shows densitometric quantification of data, expressed as fold binding with respect to background binding to GST alone (set as 1). The lower panel shows the Coomassie stained gel at the end of the binding experiment. The experiment was repeated at least three times with similar results. **C. **Schematic representation of HIV-1 Tat protein and of its mutants obtained as GST fusion proteins. The capacity of binding to hNAP-1 – see experiment in panel D – is indicated on the right side of each mutant. The dotted box corresponds to the basic domain of Tat, which binds hNAP-1. **D. **Representative GST pulldown experiment using the indicated Tat mutants (obtained as GST fusion proteins) and in vitro transcribed and translated hNAP-1 protein. The autoradiography shows the amount of hNAP-1 binding to each mutant; the histogram on top shows densitometric quantification of data, expressed as fold binding with respect to background binding to GST alone (set as 1). The lower panel shows the Coomassie stained gel at the end of the binding experiment. The experiment was repeated at least three times with similar results.

The domains within hNAP-1 and HIV-1 Tat that were responsible for the interaction were defined by in vitro GST-pulldown assays. A series of N- and C-terminal deletion mutants of hNAP-1 (Figure 3A) was expressed after fusion to GST, and incubated with ^35^S-labeled full-length HIV-1 Tat obtained by in vitro translation. All deletants lacking the N-terminus of the protein up to aa 161 bound Tat as efficiently as the full length protein; in contrast, binding was impaired when the hNAP-1 domain from residues 163 to 289 as well as the C-terminal region from residues 290 to 391 were deleted (Figure 3B). These results indicate that Tat binds two separable domains within hNAP-1, one internal from amino acids 162 to 290 and one C-terminal from residues 290 to 391.

Next we analyzed the domains of Tat responsible for the interaction with hNAP-1. GST pull-down experiments were performed using wild type Tat (101 aa), Tat72 (lacking the second exon), Tat86 (HXB2 clone), and mutated derivatives of Tat86 carrying cysteine to alanine mutations at positions 22, 25 and 27 in the cysteine-rich domain or arginine to alanine mutations at positions 49, 52, 53, 55, 56 and 57 in the basic domain (Tat86 C(22–27)A and R(49–57)A respectively); Figure 3C. These proteins, obtained as C-terminal fusions to GST, were used to pull-down ^35^S-methionine-labelled hNAP-1 obtained by in vitro transcription/translation. The results obtained demonstrated that hNAP-1 bound the basic domain of HIV-1 Tat (Figure 3D).

### hNAP-1 and Tat cooperate in the activation of HIV-1 gene expression

**Figure 4:**
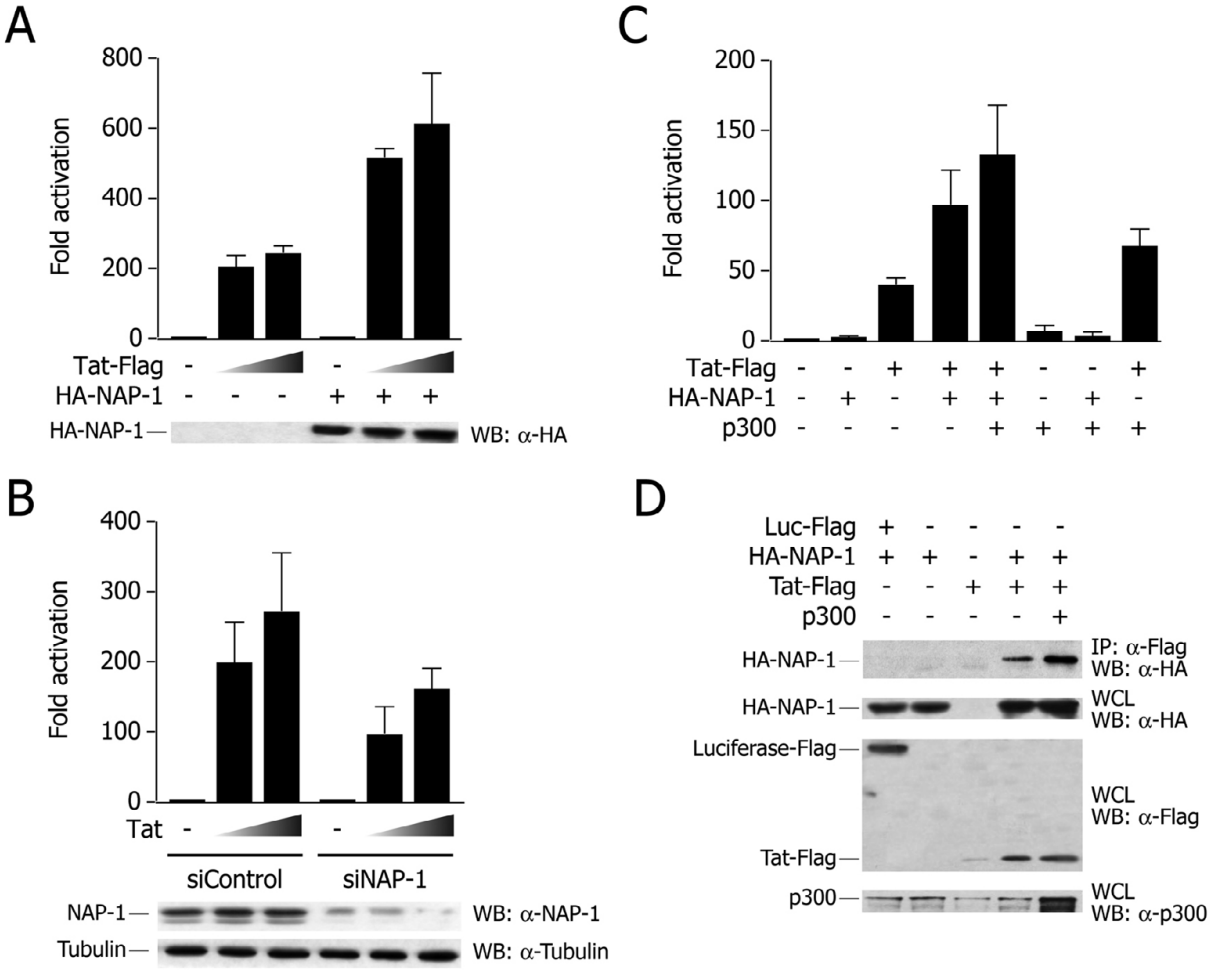
**hNAP-1 cooperates with Tat in LTR transactivation. A**. hNAP-1 synergizes with Tat in transcriptional activation. HeLa cells were cotransfected with a reporter construct containing the HIV-1 LTR upstream of the luciferase gene, and with vectors for HA-tagged hNAP-1 (100 ng) and HIV-1 Tat (5 and 25 ng), as indicated. The histogram shows mean ± s.d. of at least three independent experiments; the results are shown as fold transactivation over LTR-luciferase reporter alone. The co-expression of hNAP-1 significantly increased Tat transactivation of the LTR promoter. The western blot at the bottom shows the levels of transfected hNAP-1 protein in a representative experiment. **B. **hNAP-1 knock down decreases Tat transactivation. HeLa cells were transfected with a specific siRNA against hNAP-1 or a control siRNA, and then transfected with the LTR-luciferase reporter together with Tat (5 and 25 ng). The histogram shows mean ± s.d. of at least three independent experiments; the results are shown as fold transactivation over LTR-luciferase reporter alone. The western blot at the bottom shows the levels of endogenous hNAP-1 protein and of tubulin as a control in a representative experiment. **C. **hNAP-1, Tat and the acetyltransferase p300 synergistically activate viral transcription. HeLa cells were transfected with LTR-luciferase reporter plasmid and with vectors for HIV-1 Tat (5 ng), HA-hNAP-1 (100 ng) and p300 (100 ng), as indicated. After 24 h from transfection, luciferase assays were performed. The histogram shows mean ± s.d. of at least three independent experiments; the results are shown as fold transactivation over LTR-luciferase reporter alone. **D. **p300 enhances Tat-hNAP-1 interaction in vivo. The plasmids indicated on top of the figure were transfected into HEK 293T cells. The upper panel shows western blots with the indicated antibodies after immunoprecipitation using an anti-Flag antibody; the lower three panels show western blotting controls from whole cell lysates (WCL) from transfected cells to show the levels of expression of the transfected proteins.

One of the essential molecular events that parallel Tat-driven transcriptional activation is the modification of chromatin structure at the HIV-1 promoter [34,39]. We therefore investigated whether NAP-1 might contribute to Tat transactivation. A reporter construct containing the U3 and R sequences of the HIV-1 LTR upstream of the luciferase gene was co-transfected into HeLa cells, together with vectors for HA-tagged hNAP-1 and HIV-1 Tat. As shown in Figure 4A, hNAP-1, when co-transfected with Tat, significantly enhanced Tat-mediated transactivation of the LTR; hNAP-1 alone had no effect on promoter activity.

To test the requirement for endogenous hNAP-1 protein in Tat-mediated HIV-1 LTR transactivation, luciferase assays were performed with HeLa cells in which expression of hNAP-1 was down-regulated by RNAi. A specific siRNA oligonucleotide was designed which was able to silence ~80% of the expression of its target from 48 hours after transfection onward, as assessed by western blot analysis (Figure 4B). In hNAP-1-knock down cells, Tat transactivation of the HIV-1 LTR was significantly impaired, compared to cells treated with a control siRNA.

Collectively, the results of these experiments indicate that hNAP-1 participates in Tat-mediated control of HIV-1 gene expression.

### p300, hNAP-1 and Tat synergistically activate HIV-1 transcription

Previous work has indicated that NAP-1 interacts with the cellular transcriptional co-activator and histone acetyltransferase p300 [25-27]. Since p300 is also an essential co-factor for Tat transactivation, we investigated the effects of hNAP-1 and p300 on Tat-mediated transactivation. For this purpose, HeLa cells were transfected with an LTR-luciferase reporter plasmid and expression vectors for p300 and hNAP-1 together with Tat. As previously described [47], p300 enhanced Tat-driven transcriptional activation; when hNAP-1 was co-transfected, transcription was further increased (~3.5 fold Tat+hNAP-1+p300 over Tat alone; Figure 4C).

As shown in the co-immunoprecipitation experiment in Figure 4D, the overexpression of p300 in the same experimental conditions did not affect the levels of expression of NAP-1 or Tat proteins (as shown in the anti-Flag immunoblot). However, in cells overexpressing p300, the amount of hNAP-1 protein co-immunoprecipitating with Tat was markedly increased, a result that is consistent with the possibility that p300 might stabilize the formation of the Tat-hNAP-1 complex in vivo.

### Effect of hNAP-1 on HIV-1 infection

**Figure 5:**
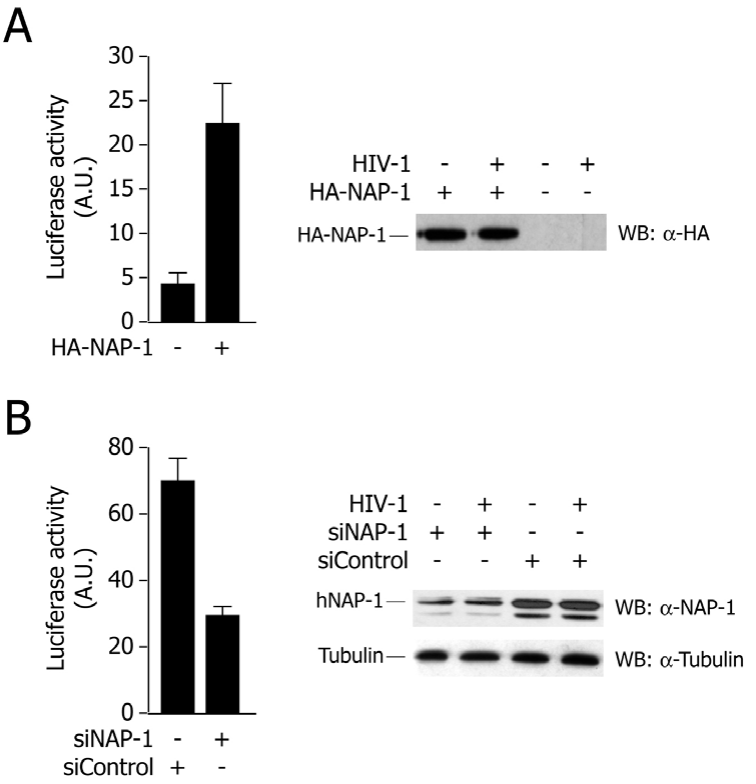
**Effect of hNAP-1 on HIV-1 infection. A**. Overexpression of hNAP-1 enhances LTR transcription upon HIV-1 infection. HeLa cells were transfected with an expression vector for HA-hNAP-1 or with a control vector, and then infected with VSG-luciferase HIV-1 vector. Luciferase activity was measured after 24 h post-infection. The mean ± s.d. of at least three different experiments is shown. The panel on the right side shows anti-HA western blottings to assess HA-hNAP-1 expression in a representative experiment. **B. **Silencing of hNAP-1 impairs LTR transcription upon HIV-1 infection. HeLa cells were treated with an siRNA directed against hNAP-1 or a control siRNA. Forty-eight hours after the beginning of siRNA treatment, cells were infected with the luciferase reported virus, and luciferase assays were performed on cell lysates 24 hours later. The mean ± s.d. of at least three different experiments is shown. The panel on the right side shows anti-hNAP-1 western blottings to assess the levels of endogenous hNAP-1 and tubulin expression in a representative experiment.

To further examine the effect of hNAP-1 on viral replication, we used an HIV vector in which a portion of nef had been replaced by the firefly luciferase gene; two frame-shifts inactivate vpr and env in this clone, thus blocking subsequent rounds of viral replication. Infectious virus, pseudotyped with VSV-G, was produced by transfections of HEK 293T cells, and used to infect HeLa cells in which hNAP-1 had been earlier either overexpressed or knocked down by RNAi. As shown in Figure 5A, the overexpression of hNAP-1 (as assessed by western blot analysis) resulted in a 5-fold increase of luciferase activity in HA-hNAP-1-transfected cells compared to mock-transfected cells. Conversely, in cells in which the levels of hNAP-1 had been reduced to <20% by RNAi, viral luciferase activity was reduced 3-fold compared to control-treated cells (Figure 5B).

Taken together, these results support the conclusion that hNAP-1 also plays an important activating role in the context of HIV-1 infection.

## Discussion

Activation of the HIV-1 LTR is a complex event involving the coordinated function of several cellular proteins acting by both releasing the negative inhibition that chromatin imposes on the promoter and inducing the recruitment of elongation-competent RNPII-containing complexes. Tat appears to exert an essential activating function for both these processes. In the last decade, a number of laboratories have reported the identification of various cellular factors that mediate Tat function. These factors fall in several broad categories, including members of the basal transcriptional machinery, among which RNAPII itself, ubiquitous transcription factors, transcriptional co-activators, histone-acetyltransferases, and others [29,59,60]. Our proteomic screening led to the identification of yet another cellular partner, hNAP-1, that appears to be essentially involved in mediating Tat function. We could confirm the interaction between Tat and hNAP-1 both in vitro and inside the cells, and demonstrate its specificity by showing that Tat was not able to co-precipitate hSET/TAF I, another member of the NAP family of proteins. The relevance of the detected interaction between Tat and hNAP-1 was further reinforced by the observations that the overexpression of hNAP-1 stimulated Tat-mediated transactivation of the LTR as well as increased HIV-1 infection. Conversely, the down-regulation of the protein by RNAi impaired both transcription and viral infection. To our knowledge, this is the first demonstration of an interaction between Tat and a histone chaperone and a first proof of the involvement of this class of proteins in the regulation of proviral transcription.

Of notice, and in contrast to our expectations, our proteomic screening did not detect several of the cellular proteins previously reported to associate with Tat and to mediate some of its functions. There are several possible explanations for this outcome. Our proteomic screening was conducted by immunoprecipitating a Flag epitope-tagged version of Tat (which was fully active transcriptionally) followed by RNase/DNase treatment, elution with a Flag peptide and resolution of Tat-associated proteins by gradient gel electrophoresis. In particular, we found that RNase treatment was essential to avoid the purification of a vast number of RNA-binding proteins unspecifically co-immunoprecipitating with Tat (data not shown). It might well be envisaged, however, that this clearing step might also affect the binding of Tat to some of its known partners, the interaction of which is strengthened by RNA bridging. In addition, RNA removal also frees the basic domain of Tat, thus rendering this region available for the interaction with hNAP-1. An additional explanation for the lack of other known Tat partners in our screening relates to the relative abundance of hNAP-1 in the cells, compared to other proteins such as p300 and P/CAF HATs, or Cyclin T1. Since our method relied on the identification of protein bands in silver-stained gels, a likely possibility is that we missed the detection of lower abundance proteins. Finally, it is worth however noting that other proteomic screenings aimed at the identification of cellular partners to other proteins also failed in identifying obvious candidates, while successfully discovering new factors essential for the function of the investigated proteins (see, among others, refs. [53,61]).

The basic region of Tat was found to bind two separable domains within hNAP-1, one internal from amino acids 162 to 290 and one C-terminal from residues 290 to 391. These domains correspond to a series of alternate α helix/β sheet regions known to be involved in the interaction with histones and other cellular proteins (see ref. [8,62] and citations therein). Of notice, the observation that Tat does not bind the highly acidic protein hSET/TAF I, another member of the NAP family with high structural and functional homology to hNAP-1 [57,58], argues in favor of a specific interaction between Tat and hNAP-1 which is not merely based on electrostatic interactions.

There is growing evidence that hNAP-1 plays important roles during transcriptional activation [21-24]. In particular, hNAP-1 and other histone chaperones both cooperate with ATP-dependent chromatin remodeling complexes [25,63] and participate in the formation of protein complexes also containing p300/CBP [25-28]. Taken together, these observations clearly suggest that hNAP-1 may serve as an interaction hub between transcriptional coactivators and chromatin. As far as p300/CBP is specifically concerned, p300 has been shown to directly bind the C-terminus of hNAP-1, namely the same region that is also involved in binding to Tat. Since the basic domain of Tat is also involved in binding to p300 [47], we cannot rule out the possibility that p300 might act as a scaffold for the simultaneous interaction with the two proteins. While further biochemical studies are clearly needed to ascertain this possibility, it is of interest to observe that the overexpression of all the three proteins together determined an increase in the levels of LTR transcription that is higher than those obtained by overexpression of either p300 or hNAP-1 alone together with Tat. In addition, expression of p300 did not affect the levels hNAP-1 or Tat proteins, but markedly increased their binding in vivo. This observation is again in favor of the possibility that p300 might exert a stabilizing role on the Tat-hNAP-1 interaction. This possibility would be consistent with the proposed function for hNAP-1 in regulating transcription in all p300-dependent promoters [27,28].

What might be the actual mechanism by which hNAP-1 might facilitate Tat transactivation? First, overexpression of hNAP-1 significantly increases the overall levels of Tat inside the cells. This result is consistent with the possibility that the interaction with hNAP-1 might increase the stability of Tat. Second, and more relevant to a specific and direct role of hNAP-1 on the LTR promoter, previous results have indicated that the acetylation of histones by p300 helps transfer histones H2A and H2B from nucleosomes to hNAP-1 [26], and that, at least in vitro, the absence of these histones correlates with increased gene activity, probably by decreasing the level of chromatin folding [64,65]. On the basis of these observations, we can speculate that hNAP-1 and p300, brought to the LTR promoter through their interaction with Tat, might cooperate in the creation of an open-chromatin environment, favorable for gene expression. Of interest, a recent genome-wide analysis in fission yeast has revealed that chromatin remodeling factors and NAP-1 colocalize within promoter regions, where they disassemble nucleosomes near the transcriptional start site, an event that is linked to changes in the levels of histone acetylation [24].

## Conclusion

In conclusion, this proteomic study reveals that the histone chaperone hNAP-1 is an important cellular factor specifically binding HIV-1 Tat. The interaction between the two proteins is involved in the regulation of Tat-mediated activation of viral gene expression, exerting a positive role on transcription. In particular, our findings indicate that HIV-1 Tat, hNAP-1 and p300 functionally cooperate to induce transcriptional activation of the HIV-1 LTR promoter.

## Methods

### Protein purification and identification

Twenty-four hours after transfection, ≈2 × 10^8 ^HEK 293T cells were washed once in phosphate-buffered saline (PBS) and lysed on ice in lysis buffer (150 mM NaCl/20 mM HEPES pH 7.9/0.5% NP-40/1 mM EDTA/1 mM DTT/protease inhibitor cocktail-Roche). The cell extract was sonicated once and then centrifuged for 15' at 14,000 rpm at 4°C. An aliquot of the cleared extract was kept as input, while the rest was incubated with 100 μl of packed and pre-equilibrated Flag M2 agarose beads overnight at 4°C. Beads were rinsed twice in lysis buffer, before treatment with DNAse I (Invitrogen, according to manufacturer's instructions) and RNAse A (150 mM NaCl/10 mM Tris HCl pH 7.5/5 mM EDTA/10 units RNAse A, for 30' at 37°C) and then washed in the same buffer three times. Immunocomplexes were eluted by adding 500 μg/ml Flag peptide (Sigma) in lysis buffer. The eluate was concentrated by standard trichloroacetic acid precipitation and resuspended in 1X sodium dodecylsulfate-polyacrylamide gel electrophoresis (SDS-PAGE) protein loading buffer. Proteins were then subjected to 6–15% gradient SDS-PAGE and then stained with silver stain. Stained proteins were excised and processed for in-gel trypsin digestion following standard protocols. The resulting peptides were extracted and purified on C18-Ziptips (Millipore) according to the manufacturer's protocol and resuspended in 10 μl of 30% methanol, 0.5% acetic acid. Protein identification was performed by the ICGEB Proteomics Facility by analyzing the purified peptides by MALDI-TOF mass spectrometry using an ABI 4800 TOF/TOF instrument (Applied Biosystems). The remaining sample was analyzed by LC-MS/MS using an LCQDeca mass spectrometer (Thermo-Finnigan).

### Cell cultures, plasmids and siRNAs

HeLa and HEK 293T cells were cultured in Dulbecco's modified Eagle's medium with Glutamax (Life Technologies, Inc.) supplemented with 10% fetal bovine serum (Life Technologies, Inc.) and gentamicin (100 μg/ml) at 37°C in a humidified 95% air-5% CO_2 _incubator.

All hNAP-1 encoding plasmids (wild type and mutants) were a kind gift by G. Steger [28]. All other plasmids used have already been described elsewhere [47,66-69].

RNA interference (RNAi) with hNAP-1 was performed against the target sequence 5' AAGGAACACGAUGAACC UAUU 3'. An siRNA targeted against the GFP RNA was used as a control (5' GGCTACGTCCAGGAGCGCACC 3'). Synthetic double-stranded RNA oligonucleotides were purchased by Dharmacon.

### Co-immunoprecipitation

For co-immunoprecipitation analyses, HEK 293T cells where transfected with the indicated plasmids using the standard calcium phosphate coprecipitation method. Twenty-four hours after transfection cells were washed once in PBS and lysed on ice in 1 ml/dish lysis buffer (150 mM NaCl/20 mM HEPES pH 7.9/0.5% NP-40/1 mM EDTA/1 mM DTT/protease inhibitor cocktail-Roche). After sonication, cleared cell extracts were incubated with pre-equilibrated Flag M2 agarose beads on a rotating wheel for 4 hours at 4°C. Beads were washed twice with 1 ml of lysis buffer, treated with DNase I (Invitrogen, according to manufacturer's instructions) and RNAse A (150 mM NaCl/10 mM Tris HCl pH 7.5/5 mM EDTA/10 units RNAse A, for 30' at 37°C) and then washed in the same buffer three times.

### Antibodies

Anti-hNAP-1 mouse monoclonal antiserum was a kind gift from Y. Ishimi [70]. Mouse monoclonal anti-Flag M2 antibody, mouse monoclonal anti-tubulin, and mouse monoclonal anti-Flag M2 agarose-conjugated beads were purchased from Sigma. Rat monoclonal anti-HA high affinity (3F10) antibody was purchased from Roche diagnostics. Rabbit polyclonal anti-GFP antibody SC8334 was purchased from Santa Cruz Biotechnology.

### Recombinant proteins

Glutathione S-transferase (GST), GST-Tat, GST-hNAP-1, GST-Tat mutants and GST-hNAP-1 mutants were prepared as already described [71]. Plasmids pcDNA3-Tat101 and pcDNA3-HA-NAP-1 were used as templates to produce the in vitro ^35^S-labeled Tat and hNAP-1 proteins, respectively, by using the TNT Reticulocyte Lysate System (Promega) according to the manufacturer's protocol.

### GST pull-down assay

GST and GST-Tat recombinant proteins immobilized on agarose beads were pre-treated with nucleases (see below). HEK293T cells were lysed in 150 mM NaCl/20 mM HEPES pH 7.9/0.5% NP-40/1 mM EDTA/1 mM DTT/protease inhibitors (Roche). Recombinant proteins and cell extracts were incubated 1 hour and 30 minutes at 4°C, and washed four times in lysis buffer.

### In vitro binding assay

To remove contaminant bacterial nucleic acids, recombinant proteins were pretreated with nucleases (0.25 U/μl DNase I and 0.2 μg/μl RNase) for 1 hour at 25°C in 50 mM Tris HCl, pH 8.0/5 mM MgCl_2_/2.5 mM CaCl_2_/100 mM NaCl/5% glycerol/1 mM DTT. Subsequently, GST fusion proteins immobilized on agarose beads were washed and resuspended in NETN buffer (20 mM Tris HCl, pH 7.5/100 mM NaCl/1 mM EDTA/0.5% NP-40/1 mM DTT/1 mM PMSF) supplemented with 0.2 mg/ml ethidium bromide to block the possible formation of non-specific interactions between residual DNA and proteins. ^35^S-labeled hNAP-1 or Tat101 proteins (400 cpm) were added and incubated at 4°C on a rotating wheel. After 90 min, bound proteins were washed twice with 0.3 ml of NETN with ethidium bromide, three times with 0.3 ml of NETN without ethidium bromide and once with 0.3 ml of 10 mM Tris HCl pH 8.0/100 mM NaCl. Finally, bound proteins were separated by electrophoresis on a 12% SDS-polyacrylamide gel. Gels were stained and fixed for 1 hour with 10% acetic acid/40% methanol/0.1% Coomassie Brilliant blue G250, and destained with 10% acetic acid/40% methanol. Dried gels were quantitated by Instant Imager (Packard).

### Luciferase assay

Reporter gene assays were performed using pLTR-luciferase plasmid as a reporter and pcDNA3-Tat101 as an effector in the presence or absence of plasmids pcDNA3-hNAP-1 and pCMV-p300. HeLa cells were transfected using Effectene Reagent (Quiagen, according to manufacturer's protocol), with 100 ng of pLTR-luciferase, 50 ng of pcDNA3-hNAP-1 and 5 or 25 ng of pcDNA3-Tat101. A Renilla luciferase expression plasmid, in which reporter gene expression was driven by the CMV promoter, was cotransfected to standardize each experiment for the efficiency of gene transfer. Cells were harvested 48 hours post transfection, and luciferase activity was measured with Luciferase assay kit (Promega). The measured activities were standardized by the activities of Renilla, and transactivation was expressed as fold activation compared with the basal activity of LTR-luciferase without effectors. All experiments were performed in duplicate and repeated at least three times.

For the transactivation experiments following RNAi, siRNAs were transfected using Oligofectamin Reagent (Invitrogen, according to manufacturer's protocol). After 36 hours from the beginning of siRNA treatment, cells were transfected with LTR-luciferase and CMV-Renilla plasmids and increasing amounts of pcDNA3-Tat101. Thirty-six hours later luciferase assays were performed on cell lysates.

In the case of infection with VSV-G-luciferase vectors, luciferase assays were performed 24 hours after the beginning of infection. For the gene-silencing experiments, cells were infected 48 hours after siRNA transfection. To normalize luciferase measures, protein concentrations in the lysates were determined with Bradford reagent (BioRad, according to manufacturer's protocol).

### Virus production and infections

To produce VSV-G-luciferase vectors, HEK 293T cells were transfected with pNL4.3-luciferase plasmid [72,73] and VSV-G encoding plasmid at a ratio 3:1, according to a standard calcium phosphate coprecipitation method. Supernatants were collected 48 hours after the beginning of transfections, centrifuged and filtered with a 45 μm syringe.

Infections with viral supernatants was carried out for 6 hours in the presence of polybrene (Sigma) at a final concentration of 5 μg/ml.

## Competing interests

The author(s) declare that they have no competing interests.

## Authors' contributions

LM, and AM carried out proteomic analysis; CV performed all other experiments. AM and ML participated in the experimental design and data analysis. MG contributed to the experimental design and coordination of the study, data analysis, as well as to writing the manuscript. All Authors have read and approved the final manuscript.
